# A cross-sectional study on the relationship between electronic cigarette and combustible cigarette use with obstructive sleep apnea among U.S. adults: result from NHANES 2015–2018

**DOI:** 10.1186/s13690-023-01083-6

**Published:** 2023-04-13

**Authors:** Hong Zhu, Meng Wu

**Affiliations:** 1 Department of Pharmacy, The Affiliated Huaian No.1 People’s Hospital of Nanjing Medical University, Huaian, Jiangsu Province 223300 China; 2 Department of Oral and Maxillofacial Surgery, The Affiliated Huaian No.1 People’s Hospital of Nanjing Medical University, 1 Huanghe West Road, Huaian, Jiangsu Province 223300 China

**Keywords:** Obstructive sleep apnea, Electronic cigarettes, Conventional cigarettes, Dual use

## Abstract

**Background:**

To explore whether the use of e-cigarettes and conventional cigarettes affects the prevalence of obstructive sleep apnea (OSA) in adults.

**Methods:**

Complete records of smoking and sleep about OSA from the 2015–2018 National Health and Nutrition Examination Survey. The adults were divided into four groups: noncurrent smokers, current electronic cigarettes (e-cigarette) users only, current conventional cigarettes (c-cigarette) users only, and dual users. OSA was assessed by three main signs and symptoms from the questionnaire. Multivariable logistic regression after adjusting for covariates was conducted to investigate the association of OSA with different smoking patterns.

**Results:**

Among the 11,248 participants, the prevalence of OSA was higher among smokers compared to non-smokers (*P* < 0.0001). In a stratified analysis of smoke manners, the results showed that an increased prevalence of OSA with c-cigarette use alone (OR = 1.38, 95% CI = 1.17–1.63) and dual-use (OR = 1.78, 95% CI = 1.37–2.32) compared to non-smoking participants, while there was no significant difference with e-cigarette use (OR = 0.84, 95% CI = 0.52–1.37). Multivariate logistic regression analysis showed the prevalence of OSA is highest in dual users (OR = 1.93, 95% CI = 1.39–2.69) than non-smokers.

**Conclusion:**

Our findings indicate the prevalence of OSA was higher in c-cigarette smokers than in non-smokers, while there was no significant difference in the prevalence of OSA between e-cigarette smokers and non-smokers. Dual users had the highest prevalence for OSA compared to c-cigarette smokers, e-cigarette smokers and non-smokers.

**Supplementary Information:**

The online version contains supplementary material available at 10.1186/s13690-023-01083-6.

## Background

Obstructive sleep apnea (OSA) is an increasingly common type of upper airway obstruction during sleep [1]. Impaired gas exchange leads to decreased oxygen saturation, hypercapnia, and sleep fragmentation, all of which have serious health consequences such as increased cardiovascular prevalence, neurocognitive dysfunction, and increased mortality [2, 3]. Currently, OSA has become one of the top public health issues, with a prevalence of about 5–15% of the overall population [4]. Therefore, it is important to understand the factors that influence OSA.

Obesity and males are the main prevalence factors for OSA, with a prevalence of 24% in middle-aged men compared to 9% in women of the same age group [5]. Obesity causes the deposition of more fat around the upper respiratory tract, changes the original ventilation condition, and directly increases the possibility of upper respiratory tract stenosis [6]. Chi et al. reported that the enlarged tongue due to fat accumulation can cause the hyoid bone to be in a posterior inferior position, narrowing the airway [7]. In addition, obesity may also affect the lung volume and increase the risk of OSA. However, the mechanism by which men are more likely to develop OSA remains unclear. The fact that more men than women smoke is probably one of the reasons for this. Currently, there is still controversy about this claim.

Epidemiological studies have showed that smoking increases the risk of OSA, and a Berlin questionnaire survey of 10,101 truck drivers found that smoking is associated with a high prevalence of OSA (odds ratio (OR) = 1.16, *P* = 0.014) [8]. Another case-control study also confirmed that smokers had a 2.5-fold higher prevalence of OSA than non-smokers or former smokers (OR = 2.5, 95% CI:1.3 ~ 4.7, *P* = 0.0049) [9]. Regarding the effect of smoking volume, a study from Thailand showed that smokers who smoked 10 cigarettes a day and had a smoking history of more than 10 years had a 4.1-fold increased risk of OSA (OR = 4.1, 95% CI:2.3 ~ 7.4) [9]. Therefore, smoking may be an independent risk factor for OSA in a dose-dependent manner.

Electronic cigarettes (e-cigarettes) are inhalable aerosol devices that are produced by electrical heating. E-cigarettes are promoted as a less harmful and more fashionable smoking tool [10]. Evidence from developed countries suggests that the popularity of e-cigarettes is rapidly increasing, especially among younger populations [11]. However, with reports of e-cigarette-related lung injury in 2019, various side effects produced by e-cigarettes in life are getting attention. To our knowledge, there are no studies on the relationship between different smoking patterns and OSA.

Understanding the impact of different smoking patterns on OSA risk can lead to better prevention and treatment. Therefore, in the present study, our primary objective was to explore if a difference exists in the prevalence of OSA according to smoking patterns, including the use of e-cigarettes and cigarettes, in a nationally representative sample of US adults.

## Methods

### Study population

**Fig. 1:**
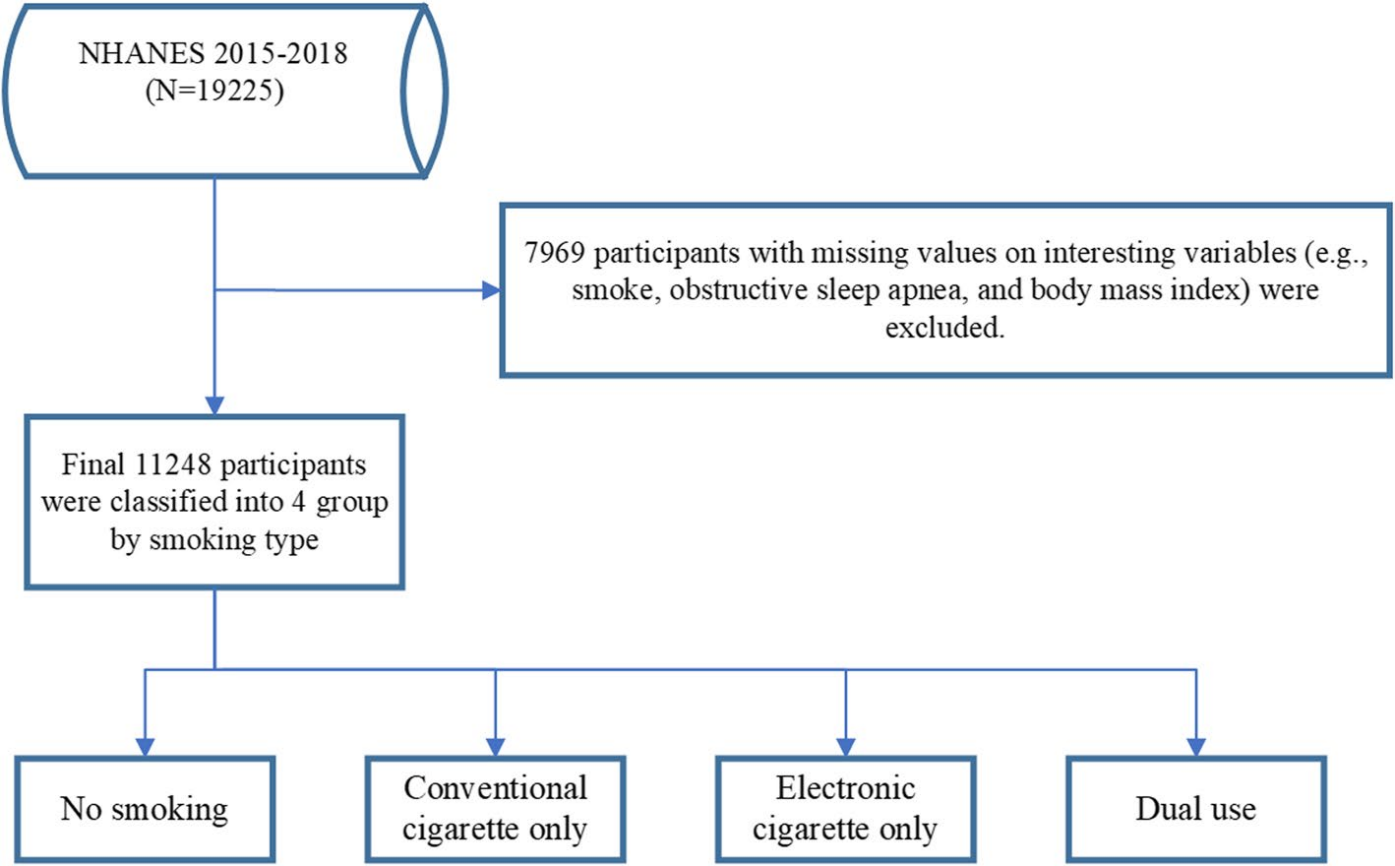
A flowchart of the participant selection process

This cross-sectional analysis was conducted using data from the 2015–2018 cycle of the National Health and Nutrition Examination Survey (NHANES). Data are deidentified, publicly available, and can be accessed at https://www.cdc.gov/nchs/nhanes. A total of 19225 participants were included in the study. A total of 7969 participants were excluded for the following reasons: missing values for OSA, smoking, and BMI. The final analytical sample included 11248 participants (Fig. 1).

### Definition of smoking status and OSA

In the NHANES 2015–2018 survey, participants were asked whether they had ever used electronic cigarettes and how many days in the past 30 days they used e-cigarettes. Participants who answered yes to the question “Have you ever used e-cigarettes?“ and used more than 0 in the past 30 days were labeled as using e-cigarettes, and others were labeled as not using e-cigarettes. Participants were also asked whether they had smoked at least 100 cigarettes in their entire lives and whether they presently smoke cigarettes every day, some days, or not at all. Participants who smoked more than 100 cigarettes in their lifetime and smoked some day or every day were defined as conventional cigarette (c-cigarette) users, and others were labeled as not using c-cigarettes. Adults were categorized into noncurrent smokers, current e-cigarette users only, current conventional cigarette users only, and dual users.

Obstructive sleep apnea (OSA) was defined as participants answering at least one of the following three questions as yes, as previously reported [12]: (1) Snoring 3 or more nights per week; (2) snorting, gasping, or stopping breathing 3 or more nights per week; or (3) feeling excessively sleepy during the day 16–30 times per month despite sleeping around 7 or more hours per night on weekdays or work nights.

### Statistical analyses

We use descriptive statistics to calculate weighted data, and the file “WTMEC2YR - Full sample 2 year MEC exam weight” in 2015–2016, 2017–2018 cycles were selected as the designated sample weight of this study. Categorical variables are calculated as unweighted frequencies and weighted estimates of overall proportions or means, and continuous variables are calculated as means ± standard errors. For continuous or categorical data, the T-test or chi-square test was utilized. Univariate analysis was used to determine the factors influencing OSA. The prevalence of OSA in different years was measured, and differences between smoking patterns were calculated. Multivariable logistic regressions were conducted to investigate the influence of smoking patterns on the prevalence of OSA. Models were adjusted for gender, age, BMI, marital status, and drinking. R 4.1.2 was used to perform all statistical analyses in this study (nhanesR package). *P* = 0.05 was used to determine statistical significance.

## Results

A total of 11,248 participants (5828 men and 5420 women) were categorized into four groups: current non-smoke (*n* = 8959), c-cigarette only (*n* = 1718), e-cigarette only (*n* = 264), and dual-use (*n* = 307). In current surveys, men and the 18–40 age group are more likely to use e-cigarettes. Smoking combustible cigarettes is still the most popular way. Participants older than 40 years old were more likely to be dual users when they smoked e-cigarettes (3.19% versus 1.33% in the 40–60 age group, and 1.40% vs. 0.48% in the 60–80 age group). Smoking increases the prevalence of OSA and the differences in the prevalence of OSA among participants with different smoking patterns (*P* < 0.0001, Table 1).

**Table 1:** Characteristics of participants according to different smoking patterns (*N* = 1,1248): result from a cross-sectional study of the United States NHANES 2015–2018

	No smoking	C-cigarette only	E-cigarette only	Dual use	*P*-value
BMI, mean (SE)	29.67 (0.19)	28.84 (0.25)	28.64 (0.76)	29.28 (0.59)	0.02
Age, mean (SE)	48.52 (0.41)	45.03 (0.59)	30.64 (1.36)	39.78 (1.19)	< 0.0001
Sex (N, Weighted %)					< 0.0001
Female	4889 (82.66)	701 (12.77)	106 (1.92)	132 (2.66)	
Male	4070 (76.40)	1017 (16.08)	158 (3.55)	175 (3.97)	
Ethnicity (N, Weighted %)					0.002
White	2849 (79.44)	643 (14.32)	98 (2.74)	143 (3.50)	
Black	1860 (75.06)	521 (19.67)	59 (2.64)	62 (2.62)	
Mexican	1488 (82.08)	199 (12.20)	45 (3.50)	33 (2.21)	
Other	2762 (82.18)	355 (12.09)	62 (2.20)	69 (3.53)	
Marital (N, Weighted %)					< 0.0001
No	3212 (73.42)	845 (18.63)	117 (4.12)	142 (3.83)	
Yes	5314 (83.31)	851 (12.35)	86 (1.42)	146 (2.92)	
Education (N, Weighted %)					< 0.0001
Less than high school	1923 (72.38)	457 (19.58)	31 (1.65)	89 (6.40)	
High school	1979 (71.23)	519 (20.11)	103 (4.83)	83 (3.84)	
More than high school	5046 (84.54)	741 (10.96)	130 (2.09)	135 (2.41)	
Annual family income (N, Weighted %)					< 0.0001
<=20,000	6588 (82.58)	1008 (12.06)	182 (2.54)	188 (2.81)	
>20,000	1672 (65.43)	565 (25.72)	59 (2.98)	98 (5.87)	
Drink (N, Weighted %)					< 0.0001
No	2168 (89.23)	185 (8.16)	26 (1.24)	26 (1.37)	
Mild	2877 (87.57)	412 (8.60)	59 (1.77)	71 (2.06)	
Moderate	1165 (76.81)	284 (16.02)	62 (4.32)	49 (2.85)	
Heavy	1110 (59.24)	540 (27.27)	91 (5.27)	132 (8.22)	
Obstructive sleep apnea (N, Weighted %)					< 0.0001
No	6271 (81.23)	1096 (13.16)	187 (2.90)	170 (2.71)	
Yes	2688 (76.19)	622 (16.98)	77 (2.29)	137 (4.54)	

Table 2 shows the associations between e-cigarette and cigarette smoking status and OSA. Univariate analysis showed an increased prevalence of OSA with c-cigarette use alone (OR = 1.38, 95% CI = 1.17–1.63) and dual-use (OR = 1.78, 95% CI = 1.37–2.32) compared to non-smoking participants, while there was no significant difference with e-cigarette use (OR = 0.84, 95% CI = 0.52–1.37). In addition, gender, age, BMI, marriage, and alcohol consumption were also independent influences on OSA. There was no significant association between education level, average household income, ethnicity, and OSA.

**Table 2:** Univariate analysis of association of smoking with obstructive sleep apnea: result from a cross-sectional study of the United States NHANES 2015-2018

	obstructive sleep apnea (OR (95% CI))	*P*-value
BMI	1.07 (1.06,1.08)	**< 0.01**
Age	1.01 (1.01,1.01)	**< 0.01**
Sex		
Female	Reference	-
Male	1.36 (1.18,1.58)	**< 0.01**
Ethnicity		
White	Reference	-
Black	1.00 (0.86,1.16)	0.99
Mexican	0.94 (0.78,1.14)	0.54
Other	0.84 (0.71,1.00)	0.05
Marital		
No	Reference	-
Yes	1.34 (1.18,1.53)	**< 0.01**
Education		
Less than high school	Reference	-
High school	1.04 (0.89,1.21)	0.60
More than high school	1.00 (0.87,1.14)	0.95
Annual family income		
<=20,000	Reference	-
>20,000	1.01 (0.90,1.13)	0.90
Drink		
No	Reference	-
Mild	1.23 (1.03,1.46)	**0.02**
Moderate	1.14 (0.93,1.39)	0.20
Heavy	1.32 (1.10,1.59)	**< 0.01**
Smoking		
No smoking	Reference	-
C-cigarette only	1.38 (1.17,1.63)	**< 0.01**
E-cigarette only	0.84 (0.52,1.37)	0.48
Dual use	1.78 (1.37,2.32)	**< 0.01**

The weighted estimate of the prevalence of OSA for different smoking patterns in different years is presented in Table 3. The participants with the highest occurrence of OSA in different years were dual users, with 42.30% (2015–2016) and 44.68% (2017–2018), respectively. Participants with the lowest OSA prevalence in 2015–2016 were non-smokers (28.79%), while in 2017–2018 were e-cigarette smokers (17.26%).

**Table 3:** Trends in the prevalence of obstructive sleep apnea by different smoking patterns and year: result from a cross-sectional study of the United States NHANES 2015–2018

	Prevalence of OSA, % (95%CI)				Differences in the prevalence of OSA, % (95%CI)			
	No smoking	C-cigarette only	E-cigarette only	Dual use	C-cigarette vs. No smoking	E-cigarette vs. No smoking	E-cigarette vs. C-cigarette	Dual use vs. C-cigarette
2015–2016	28.79 (27.02–30.56)	37.92 (33.14–42.69)	41.36 (29.78–52.93)	42.30 (34.88–49.72)	9.12 (3.68–14.57)	12.56 (0.77–24.36)	3.44 (-10.26- 17.13)	4.385 (-5.29- 14.06)
2017–2018	31.46 (28.79–34.13)	36.56 (32.97–40.16)	17.26 (5.01–29.52)	44.68 (36.04–53.33)	5.11 (0.38–9.83)	-14.12 (-27.21- -1.18)	-19.3 (-31.41–7.19)	8.12 (-0.53- 16.76)

Table 4 presents a multivariate logistic regression analysis summarizing the effect of different smoking patterns on the prevalence of OSA. Adjusted for gender, age, BMI, marital status, and drink, the ORs of OSA were 1.40 (95% CI: 1.18–1.67) in c-cigarette users, 1.14 (95% CI: 0.65–1.99) in e-cigarette users, and 1.94 (95% CI: 1.40–2.69) in dual users, compared to non-users.

**Table 4:** Adjusted multinomial logistic regression of Smoking with obstructive sleep apnea: result from a cross-sectional study of the United States NHANES 2015–2018

Regression model	No smoking	C-cigarette only		E-cigarette only		Dual use		*p* for trend
		OR (95% CI)	*P*	OR (95% CI)	*P*	OR (95% CI)	*P*	
Model 1	Reference	1.38 (1.17,1.63)	< 0.001	0.84 (0.52,1.37)	0.48	1.78 (1.37,2.32)	< 0.01	< 0.01
Model 2	Reference	1.41 (1.19,1.67)	< 0.001	0.99 (0.60,1.66)	0.98	1.93 (1.49,2.50)	< 0.01	< 0.01
Model 3	Reference	1.52 (1.29,1.77)	< 0.001	1.01 (0.62,1.66)	0.95	2.00 (1.52,2.63)	< 0.01	< 0.01
Model 4	Reference	1.40 (1.18,1.67)	< 0.001	1.14 (0.65,1.99)	0.63	1.94 (1.40,2.69)	< 0.01	< 0.01

Model 1: Unadjusted

Model 2: Adjusted for gender, and age

Model 3: Adjusted for gender, age, and BMI

Model 4: Adjusted for gender, age, BMI, marital, and drink

## Discussion

Our current findings, based on a survey of a representative US population, demonstrate that smoking in adults is associated with a higher prevalence of OSA. Additionally, among adults with different smoking patterns, a significant association between c-cigarette use only and a high prevalence of OSA was observed. However, e-cigarette-only smoking did not affect the prevalence of OSA compared to non-smoking adults. In addition, we found the highest prevalence of OSA in participants who smoked both e-cigarettes and conventional cigarettes.

OSA is an unfavorable factor in multiple systems of the body, including the metabolic system, the neuropsychiatric system, and the cardiovascular system. The well-known etiology of OSA is primarily caused by recurrent episodes of the partial or complete collapse of the upper airway during sleep. OSA is increasingly being recognized as a significant cause of increased mortality [13]. It is noteworthy that despite the great progress made in the past in the recognition of OSA, 70–80% are still undiagnosed [14, 15]. Since patients are often unaware of their symptoms while sleeping, they are usually detected by their bed partners or family members, missing the best time for early intervention, which can lead to serious complications. Therefore, understanding the prevalence factors for OSA is crucial for the population to receive early diagnosis and treatment.

Recent research suggests that participants who smoke are more likely to snore and develop OSA [16]. A recent meta-analysis revealed that secondhand smoke exposure has been independently associated with OSA [17]. Cigarette smoke-induced airway inflammation and injury alter the mechanical and neurological properties of the upper airway and increase its collapsibility during sleep [13]. Overall, these cases support the view that participants who traditionally smoked were significantly associated with OSA. Our results are consistent with previous studies showing that smokers have a higher prevalence of OSA than non-smokers.

Recently, the use of e-cigarettes has been on the rise among young people and adults. In a previous study, 43% of young people reported that e-cigarettes were less harmful than cigarettes [18]. Even e-cigarettes are sometimes considered a quit-smoking tool. However, with reports of e-cigarette-related lung injury in 2019, various side effects produced by e-cigarettes in life are getting attention. To the best of our knowledge, there are no studies on the effects of different smoking patterns on OSA. In the present study, we planned to investigate whether e-cigarette users were positively associated with a higher prevalence of OSA. We found that e-cigarette smokers did not increase the prevalence of OSA (OR = 0.84, 95% CI = 0.52–1.37). Interestingly, the prevalence of e-cigarettes for OSA was different in the results of different years. The prevalence of OSA among e-cigarette smoking participants in 2015–2016 was 28.79%, which was higher than that of non-smokers. While the lowest OSA prevalence rate was 17.26% among e-cigarette smokers in 2017–2018. Similarly, the prevalence of OSA in e-cigarette users was not significantly different from that in non-smokers in a fully adjusted analysis. The prevalence of OSA is higher among e-cigarette smokers and dual users than among non-smokers. The difference in the prevalence of OSA among e-cigarette users between years may be due to the population bias in different years. More extensive population surveys are needed to verify.

The relationship between smoking and OSAS may be caused by the following factors: (1) smoking changes the sleep structure of the patient and reduces sleep stability; (2) nicotine in tobacco leaves the upper respiratory tract muscles loose, the nerve reaction weakened, and the respiratory tract collapses easily; (3) nicotine increases the threshold of sleep-wake; and (4) smoking stimulates the mucous membrane of the upper respiratory tract and aggravates the inflammatory reaction of the respiratory tract [19]. In addition, smoking causes edema and thickening of the hanging mucous membrane, leading to narrowing of the upper respiratory tract and oropharynx, which may also be the mechanism of a severe increase in OSAS [20]. In addition, studies have shown that 35% of OSA patients smoke, while only about 18% of non-OSA patients smoke [9]. Although no clinical studies have been conducted to assess if OSA will aggravate tobacco addiction, some studies have shown that some patients can relieve clinical manifestations of OSA such as drowsiness, anxiety, or depression by smoking, and smoking may become one of the methods for self-treatment of daytime symptoms in OSA patients [21]. At the same time, a small sample study showed that 60% of 65 patients with OSA successfully quit smoking during 5 years of continuous positive airway pressure treatment [22]. However, the specific mechanism still needs further study.

Despite the novel findings of this study from a US population sample, it has several limitations. First, records of related situations were a self-report of participants’ recollections, and thus, memory bias may exist. Second, the present study could not exclude all confounding factors, which would have an impact on the results, such as the effect of different geographical locations and lifestyles on OSA. Third, the differences in gender and age distribution between the two groups may affect the results.

## Conclusion

Our findings indicate the prevalence of OSA was higher in c-cigarette smokers than in non-smokers, while there was no significant difference in the prevalence of OSA between e-cigarette smokers and non-smokers. Dual users had the highest prevalence for OSA compared to c-cigarette smokers, e-cigarette smokers and non-smokers.

## Supplementary Information

**Additional file 1. **Aims and Scope statement.

## Data Availability

The data are available from the website (https://www.cdc.gov/nchs/nhanes/index.htm).

## Abbreviations

OSA: Obstructive sleep apnea
e-cigarette: Electronic cigarettes
c-cigarette: Conventional cigarettes

## References

[1] Jordan AS, McSharry DG, Malhotra A (2014). Adult obstructive sleep apnoea. Lancet (London England).

[2] McNicholas WT, Bonsigore MR (2007). Sleep apnoea as an independent risk factor for cardiovascular disease: current evidence, basic mechanisms and research priorities. Eur Respir J.

[3] Yaggi HK, Concato J, Kernan WN, Lichtman JH, Brass LM, Mohsenin V (2005). Obstructive sleep apnea as a risk factor for stroke and death. N Engl J Med.

[4] Young T, Peppard PE, Gottlieb DJ (2002). Epidemiology of obstructive sleep apnea: a population health perspective. Am J Respir Crit Care Med.

[5] Young T, Palta M, Dempsey J, Skatrud J, Weber S, Badr S (1993). The occurrence of sleep-disordered breathing among middle-aged adults. N Engl J Med.

[6] Schwartz AR, Patil SP, Squier S, Schneider H, Kirkness JP, Smith PL (2010). Obesity and upper airway control during sleep. J Appl Physiol (Bethesda Md: 1985).

[7] Chi L, Comyn FL, Mitra N, Reilly MP, Wan F, Maislin G, Chmiewski L, Thorne-FitzGerald MD, Victor UN, Pack AI (2011). Identification of craniofacial risk factors for obstructive sleep apnoea using three-dimensional MRI. Eur Respir J.

[8] Moreno CR, Carvalho FA, Lorenzi C, Matuzaki LS, Prezotti S, Bighetti P, Louzada FM, Lorenzi-Filho G (2004). High risk for obstructive sleep apnea in truck drivers estimated by the Berlin questionnaire: prevalence and associated factors. Chronobiol Int.

[9] Kashyap R, Hock LM, Bowman TJ (2001). Higher prevalence of smoking in patients diagnosed as having obstructive sleep apnea. Sleep Breath.

[10] Grana R, Benowitz N, Glantz SA (2014). E-cigarettes: a scientific review. Circulation.

[11] Dockrell M, Morrison R, Bauld L, McNeill A (2013). E-cigarettes: prevalence and attitudes in Great Britain. Nicotine & tobacco research: official journal of the Society for Research on Nicotine and Tobacco.

[12] Cavallino V, Rankin E, Popescu A, Gopang M, Hale L, Meliker JR (2022). Antimony and sleep health outcomes: NHANES 2009–2016. Sleep health.

[13] Punjabi NM (2008). The epidemiology of adult obstructive sleep apnea. Proc Am Thoracic Soc.

[14] Young T, Evans L, Finn L, Palta M (1997). Estimation of the clinically diagnosed proportion of sleep apnea syndrome in middle-aged men and women. Sleep.

[15] Kapur V, Strohl KP, Redline S, Iber C, O’Connor G, Nieto J (2002). Underdiagnosis of sleep apnea syndrome in U.S. communities. Sleep & breathing = Schlaf & Atmung.

[16] Khoo SM, Tan WC, Ng TP, Ho CH (2004). Risk factors associated with habitual snoring and sleep-disordered breathing in a multi-ethnic asian population: a population-based study. Respir Med.

[17] Chang CW, Chang CH, Chuang HY, Cheng HY, Lin CI, Chen HT, Yang CC (2022). What is the association between secondhand smoke (SHS) and possible obstructive sleep apnea: a meta-analysis. Environ health: global access Sci source.

[18] Bernat D, Gasquet N, Wilson KO, Porter L, Choi K (2018). Electronic cigarette harm and benefit perceptions and use among Youth. Am J Prev Med.

[19] Krishnan V, Dixon-Williams S, Thornton JD (2014). Where there is smoke there is sleep apnea: exploring the relationship between smoking and sleep apnea. Chest.

[20] Kim KS, Kim JH, Park SY, Won HR, Lee HJ, Yang HS, Kim HJ (2012). Smoking induces oropharyngeal narrowing and increases the severity of obstructive sleep apnea syndrome. J Clin Sleep Med.

[21] Schrand JR (1996). Is sleep apnea a predisposing factor for tobacco use?. Med Hypotheses.

[22] Chaouat A, Weitzenblum E, Kessler R, Oswald M, Sforza E, Liegeon MN, Krieger J (1997). Five-year effects of nasal continuous positive airway pressure in obstructive sleep apnoea syndrome. Eur Respir J.

