# Supplementary material for: A cross-sectional study on the relationship between electronic cigarette and combustible cigarette use with obstructive sleep apnea among U.S. adults: result from NHANES 2015–2018
Source: Arch Public Health. 2023 Apr 13;81:54. doi: 10.1186/s13690-023-01083-6 (PMC10099817; doi:10.1186/s13690-023-01083-6)
Supplement: Supplementary file 1 — Additional file 1. Aims and Scope statement. [file 13690_2023_1083_MOESM1_ESM.docx]

**Aims and Scope statement**

**1.      What is known**

Obstructive sleep apnea (OSA) is an increasingly common type of upper airway obstruction during sleep. Currently, OSA has become one of the top public health issues, with a prevalence of about 5 - 15% of the overall population. Therefore, it is important to understand the factors that influence OSA. Currently, whether smoking causes OSA remains controversial. Wetter et al. found that smokers were twice as likely to develop sleep apnea as non-smokers. Conversely, a notable study reported that men and women have a similar risk of sleep apnea due to smoking compared to non-smokers.

E-cigarettes are promoted as a less harmful and more fashionable smoking tool. Evidence from developed countries suggests that the popularity of e-cigarettes is rapidly increasing, especially among younger populations. However, with reports of e-cigarette-related lung injury in 2019, various side effects produced by e-cigarettes in life are getting attention.

**2.      What does the study adds**

Understanding the impact of different smoking patterns on OSA risk can lead to better prevention and treatment. To our knowledge, there are no studies on the relationship between different smoking patterns and OSA. Therefore, in the present study, our primary objective was to explore if a difference exists in the risk of OSA according to smoking patterns, including the use of e-cigarettes and combustible cigarettes. In order to reduce selection bias, we chose a retrospective study based on the National Health and Nutrition Examination Survey (NHANES).

**3.      What are implications for clinical practice, public health and / or research.**

Our findings indicate the incidence of OSA was higher in combustible cigarette smokers than in non-smokers, while there was no significant difference in the incidence of OSA between e-cigarette smokers and non-smokers. Dual users were at the highest risk for OSA among those who smoked in different manners.

The findings of our study imply that we need to pay attention to the risk of OSA in the smoking population. Although our results show that e-cigarette use alone does not increase the risk of OSA, given the limited number of e-cigarette users, further studies are needed.
